# The Role of Sirtuins in Kidney Diseases

**DOI:** 10.3390/ijms21186686

**Published:** 2020-09-12

**Authors:** Yu Ah Hong, Ji Eun Kim, Minjee Jo, Gang-Jee Ko

**Affiliations:** 1 Department of Internal Medicine, College of Medicine, The Catholic University of Korea, Daejeon St. Mary Mary’s Hospital, Daejeon 34943, Korea; amorfati@catholic.ac.kr; 2 Department of Internal Medicine, Korea University College of Medicine, Korea University Guro Hospital, Seoul 08308, Korea; beeswaxag@naver.com (J.E.K.); minjeeyoyo@naver.com (M.J.)

**Keywords:** sirtuins, kidney, acute kidney injury, diabetic nephropathy, chronic kidney disease, aging kidney

## Abstract

Sirtuins (SIRTs) are class III histone deacetylases (HDACs) that play important roles in aging and a wide range of cellular functions. Sirtuins are crucial to numerous biological processes, including proliferation, DNA repair, mitochondrial energy homeostasis, and antioxidant activity. Mammals have seven different sirtuins, SIRT1–7, and the diverse biological functions of each sirtuin are due to differences in subcellular localization, expression profiles, and cellular substrates. In this review, we summarize research advances into the role of sirtuins in the pathogenesis of various kidney diseases including acute kidney injury, diabetic kidney disease, renal fibrosis, and kidney aging along with the possible underlying molecular mechanisms. The available evidence indicates that sirtuins have great potential as novel therapeutic targets for the prevention and treatment of kidney diseases.

## 1. Introduction

Sirtuins (SIRTs) are a family of nicotinamide adenine dinucleotide (NAD)^+^-dependent class III histone deacetylases (HDACs) that use the coenzyme NAD^+^ to deacetylate lysine residues in histone and non-histone proteins. Due to their ability to target post-translational acyl modifications of various cellular substrates, sirtuins are crucial to numerous biological processes including proliferation, DNA repair, mitochondrial energy homeostasis, and antioxidant activity [1]. Silent information regulator 2 (SIR2) was the first sirtuin discovered and was originally found in *Saccharomyces cerevisiae* [2]. SIR2 is critical for transcriptional silencing in budding *S. cerevisiae* and extension of the lifespan of mother cells through suppression of toxic recombinant DNA circle formation, as well as other processes including the management of molecules damaged by oxidative stress [3]. Seven different sirtuin proteins localized to different subcellular compartments have been identified in mammals [4]. SIRT1 and SIRT2 are distributed in both the nucleus and cytoplasm. SIRT3, SIRT4, and SIRT5 are mainly present in the mitochondria, while SIRT6 and SIRT7 are primarily found in the nucleus [1,5]. The diverse biological functions of the various sirtuins are attributed to differences in subcellular localization, expression profiles, and cellular substrates.

## 2. Molecular Targets of Sirtuins

SIRT1, as the first sirtuin identified in mammals, has been the most widely investigated. Although SIRT1 is mainly present in the nucleus, it can translocate into the cytoplasm under specific conditions, such as ischemic stress or embryonic development [6,7]. In the kidney, SIRT1 is widely expressed in tubular cells and podocytes [8]. At the cellular level, SIRT1 may play important roles in several biological processes, including energetic homeostasis [9], apoptosis [10], mitochondrial biogenesis [11], and autophagy [12]. SIRT1 mediates the longevity effect of caloric restriction through regulation of glucose and lipid metabolism [9,13]. Caloric restriction increases the NAD^+^/NADH ratio and subsequently SIRT1 activity [14]. Using the coenzyme NAD^+^, SIRT1 enhances chromatin silencing and transcriptional repression through deacetylation of histones [15]. SIRT1 can directly deacetylate histone lysines H4K16 (H4 Lys16), H3K9 (H3 Lys9), H3K14 (H3 Lys14), and H1K26 (H1 Lys26) upon recruitment to chromatin [16]. SIRT1 acts as the key transcriptional modulator of cell survival via regulation of p53 [17], nuclear factor-κB (NF-κB) p65 [18], signal transducer and activator of transcription 3 (STAT3) [19], and the Forkhead boX class O (FoxO) family [20]. SIRT1 regulates the cellular response to hypoxic stress through deacetylation of hypoxia-induced factor 1α (HIF-1α) [21,22]. SIRT1 also activates peroxisome proliferator-activated receptor gamma (PPARγ) through deacetylation of PPARγ coactivator-1α (PGC-1α) [23,24]. In addition, SIRT1 regulates cellular homeostasis through its activation of adenosine monophosphate-activated protein kinase (AMPK) via liver kinase B1 (LKB1) and inhibition of the mammalian target of rapamycin (mTOR) [25,26]. SIRT1 also accelerates deacetylation and nuclear translocation of β-catenin and regulates the Wnt/β-catenin transcriptional pathway [27].

SIRT2 is a cytoplasmic sirtuin that is co-localized with microtubules and deacetylates the major component of microtubules [28]. However, SIRT2 has also been observed in the nucleus and mitochondria, and its translocation to the nucleus may indicate an important epigenetic role [29]. SIRT2 has been shown to deacetylate several substrates including histone lysines H4K16 [30], H3K56 (H3 Lys56) [31], α-tubulin [28], PR-Set7 [32], phosphoenolpyruvate carboxykinase 1 (PEPCK1) [33], NF-κB p65 [34], and FoxO family proteins [35,36], and is also involved in the regulation of the cell cycle, DNA repair, and stress responses [30]. SIRT2 regulates binding of p65 to the promoters of anti-inflammatory chemokines such as C-X-C motif chemokine ligand 2 (CXCL2) and CC motif chemokine ligand 2 (CCL2) [34]. SIRT2 also regulates insulin-induced protein kinase B (PKB/AKT) activation via constitutive phosphoinositide 3-kinase (PI3K) activity, and this regulation is involved in AMPK-dependent SIRT2 phosphorylation [37]. In addition, SIRT2 is a central regulator of the defense mechanism against reactive oxidative species (ROS) through FoxO3a deacetylation [36]. In the kidney, SIRT2 is mainly expressed in proximal epithelial tubular cells [34].

SIRT3 is the best characterized among mitochondrial sirtuins. SIRT3 is normally localized inside the mitochondria, but can translocate into the nucleus under stressful conditions, along with overexpression of SIRT5 [38]. SIRT3 is a NAD^+^-dependent deacetylase in mitochondria that regulates energy metabolism; the anti-oxidative defense system; lipid metabolism; and longevity via deacetylation of various substrates including acetyl-coenzyme A synthetase 2 (ACSS2) [39], glutamate dehydrogenase (GDH) [40], and superoxide dismutase 2 (SOD2) [41]. SIRT3 also reduces levels of ROS through regulation of NF-κB [42], AMPK [43], PGC-1α [44], and FoxO3a [45]. SIRT3 regulates the acetylation status of multiple proteins to improve function, including those associated with fatty acid oxidation, ketogenesis, amino acid catabolism, and urea and tricarboxylic acid cycles, in addition to mitochondrial regulation [40,46]. In the kidney, SIRT3 has been described as a crucial regulator of mitochondrial dynamics in proximal epithelial tubular cells [47].

SIRT4, the second mitochondrial sirtuin, has been observed in the mitochondrial matrix where it binds to proteins in similar pathways to those regulated by SIRT3. SIRT4 exhibits NAD^+^-dependent adenosine diphosphate (ADP)-ribosyltransferase activity and inhibits GDH activity through ADP-ribosylation [48]. SIRT4 is also a lysine deacetylase and can remove multiple derivatives of methylglutaryl from lysine residues to regulate leucine metabolism [49]. However, reduction of SIRT4 under conditions of nutrient deprivation coordinates the use of fatty acids as an energy source, suggesting that SIRT4 might antagonize the dietary restriction-mediated effects of SIRT1 and SIRT3. SIRT4 also suppresses fatty acid oxidation through inhibition of malonyl CoA decarboxylase (MCD) [50], peroxisome proliferator-activated receptor α (PPARα) [51], and AMPK signaling [52]. SIRT4 also acts as a tumor suppressor, regulating the cellular metabolic response to DNA damage via repression of glutamine metabolism [53]. However, little is known about the role of SIRT4 in the kidney [54].

SIRT5, the third mitochondrial sirtuin, was thought to localize primarily to mitochondria [55]. However, several studies have demonstrated that SIRT5 is also present in the cytosol, peroxisomes, and nucleus [56,57,58]. SIRT5 was initially described as a mitochondrial deacetylase, but it has minimal deacetylase activity [59]. Recent studies have reported that SIRT5 shows distinct affinity for negatively charged acyl lysine modifications, and acts as a prominent cellular desuccinylase, demalonylase, and deglutarylase, thereby regulating various metabolic pathways [59,60]. Pathway analysis identified multiple target pathways of SIRT5 including fatty acid oxidation, branched-chain amino acid catabolism, the citric acid cycle, adenosine triphosphate (ATP) synthesis, oxidative phosphorylation (OXPHOS), stress responses, ketogenesis, and pyruvate metabolism [61]. The absence of SIRT5 led to hypersuccinylation of mitochondrial proteins in the kidney [62,63] and increased blood ammonia levels [64], suggesting that SIRT5 may play roles in renal and liver metabolism. A recent study revealed that SIRT5 is highly expressed in proximal epithelial tubular cells [65]. However, the function of SIRT5 in the kidney remains poorly understood.

SIRT6 is predominantly a nuclear protein and deacetylates diverse proteins, including H3K9 and H3K56 [66]. SIRT6 exhibits both deacetylase and mono-ADP-ribosyltransferase activities [67]. Like other sirtuins, SIRT6 regulates longevity via deacetylation of several transcription factors associated with DNA repair, glucose and lipid metabolism, cellular senescence, and inflammation [68,69,70,71]. SIRT6 also functions as a corepressor of HIF-1α, suppressing glucose uptake and glycolysis [70]. In the kidney, SIRT6 plays an important role in podocyte injury and renal fibrosis in podocytes and proximal epithelial tubular cells [72,73,74].

SIRT7 is localized to the nucleus and has NAD^+^-dependent HDAC activity [75]. SIRT7 appears to exhibit relatively weak and substrate-specific deacetylase activity. The known deacetylation substrates of SIRT7 are only H3K18 (H3 Lys18), p53, polymerase-associated factor 53 (PAF53), the nucleolar protein nucleophosmin (NPM1), and GA-binding protein-β-1 (GABP-β1) [76]. SIRT7 deficiency is associated with a high mutation rate, increased sensitivity to DNA damage, and apoptosis. These findings suggest that SIRT7 regulates genome stability through its effects on transcriptional regulation, DNA replication, and DNA repair [77,78]. In the kidney, SIRT7 is expressed in proximal tubules and collecting ducts [79]. However, the role of SIRT7 in kidney injury remains unclear.

## 3. The Role of Sirtuins in Renal Disease

### 3.1. Sirtuins in Acute Kidney Injury

Interest in the role of sirtuins in the pathophysiology of various renal diseases has grown recently. Given the tubular expression of sirtuins and their modulating effects on oxidative stress, inflammation, and mitochondrial dysfunction, they are likely to be involved in the pathogenesis of acute kidney injury (AKI) [80].

#### 3.1.1. Sirtuins in Ischemia/Reperfusion-Induced Kidney Injury

Greater susceptibility to injury caused by ischemia/reperfusion (I/R) in aging kidneys raises the possibility that sirtuins may play a role in the pathogenesis of I/R-induced kidney injury. SIRT1 overexpression was associated with enhanced resistance to kidney injury after I/R, whereas the loss of one SIRT1 allele aggravated kidney injury following I/R [81]. SIRT1 attenuated I/R-induced kidney injury along with activation of anti-oxidant pathways such as nuclear factor erythroid 2-related factor 2 (Nrf2)/heme oxygenase-1 (HO-1) signaling [82] and reduction of p53 expression and apoptosis [81]. SIRT1 also attenuated I/R-induced kidney injury by stimulating mitochondrial biogenesis. Treatment with SRT1720, an SIRT1 activator, restored renal ATP levels through reduction of mitochondrial mass, nitrosative stress, and inflammation, leading to attenuation of I/R-induced kidney injury [11]. Promotion of mitochondrial biogenesis and PGC-1α activation by activators of SIRT1 has also been proposed as repair mechanisms after I/R-induced kidney injury [83].

I/R injury was found to increase SIRT3 expression in the kidney. Given that SIRT3 is mainly localized in the mitochondrial matrix, SIRT3 may affect the course of I/R-induced kidney injury, which is associated with mitochondrial dysfunction. SIRT3 overexpression was found to confer renal protection via the suppression of superoxide generation [84]. Reduced expression of SIRT3 was associated with increased severity of I/R-induced kidney injury, while restoration of SIRT3 reversed this damage by modulating mitochondrial homeostasis through the AMPK/PGC-1α pathway [85]. A recent study reported that SIRT5 was highly expressed in both the mitochondria and peroxisomes of proximal tubular cells, but the opposite trend was observed in I/R-induced kidney injury compared to other types of sirtuins, as the loss of SIRT5 function led to renoprotective effects after I/R injury; shifting of fatty acid oxidation from the mitochondria to the peroxisome under the control of SIRT5 was suggested as the underlying mechanism. In contrast, SIRT6 expression was negatively correlated with the degree of hypoxia-induced tubular cell injury and inflammation of tubular epithelial cells [86]. The differing effect of each sirtuin on I/R-induced kidney injury should be elucidated through future researches.

#### 3.1.2. Sirtuins in Cisplatin-Induced Acute Kidney Injury

Cisplatin-induced kidney injury decreases mitochondrial number and function, and also increases production of ROS. Given the core role of sirtuins in mitochondrial biogenesis and integrity, the role of sirtuins has been more extensively studied in cisplatin-induced AKI compared to other causes of AKI. Renal tubule-specific SIRT1 transgenic mice showed attenuation of functional and histological measures of kidney injury after cisplatin treatment, which was attributed to decreases in cisplatin-induced oxidative stress and apoptosis [87]. Pharmacologic activation of SIRT1 was also associated with the attenuation of cisplatin-induced AKI via modulation of oxidative stress and inflammation through the NF-κB and p53 signaling pathways [88,89,90]. Recent studies have highlighted the renoprotective activity of SIRT3 in cisplatin-induced AKI through modulation of mitochondrial dysfunction. Loss of SIRT3 function in mice led to aggravation of renal function deterioration after cisplatin treatment, and attenuation of cisplatin-induced kidney injury through pharmacologic activation of SIRT3, which was observed in wild-type mice, did not occur in SIRT3-deficient mice. In tubular cells, reduced SIRT3 expression after cisplatin treatment resulted in mitochondrial fragmentation, while activation of SIRT3 expression reversed the injury and preserved mitochondrial integrity [91]. The renoprotective role of SIRT3 in cisplatin-induced AKI through modulation of mitochondrial dysfunction has been further demonstrated in other studies [92,93]. One of nuclear sirtuins, SIRT6 knockout mice exhibited aggravation of cisplatin-induced kidney injury, while SIRT6 attenuated renal inflammation and apoptosis by deacetylating H3K9 and inhibiting expression of extracellular-signal-regulated kinase (ERK)-1/2 [94].

In contrast to SIRT1, SIRT3, and SIRT6, the absence of SIRT2 and SIRT7, not their overexpression, significantly ameliorated cisplatin-induced AKI by decreasing inflammation and apoptosis through modulation of p38 and c-Jun N-terminal kinase (JNK) [79,90]. Conflicting findings have been reported regarding the role of SIRT5 in cisplatin-induced AKI. In one study of renal tubular cells, SIRT5 overexpression was found to attenuate cisplatin-induced apoptosis and mitochondrial injury through regulation of Nrf2/HO-1 and B-cell lymphoma 2 (Bcl-2) [95]. However, another study reported that deficiency of SIRT5 function in mice significantly improved renal function and tubular damage in cisplatin-induced AKI through peroxisomal fatty acid oxidation of proximal tubules [65]. The exact role of SIRT5 in cisplatin-induced AKI requires further elucidation.

#### 3.1.3. Sirtuins in Other Types of Acute Kidney Injury

The immunomodulatory function of most sirtuins has been shown to relieve sepsis-induced AKI. Overexpression of SIRT1 and SIRT6 was associated with alleviation of tubular injury induced by lipopolysaccharide treatment in an animal model of cecal ligation and puncture [96,97]. Similar results were obtained from sepsis-induced AKI model treated with resveratrol, a sirtuin activator [98]. The renoprotective effect of sirtuins in sepsis-induced AKI was accompanied by suppression of inflammasome activation and promotion of autophagy. However, consistent with cisplatin-induced AKI, loss of SIRT2 function in mice improved renal function and renal tubular injury after lipopolysaccharide treatment [34].

The role of sirtuins has been also demonstrated in contrast-induced nephropathy (CIN), which is the third leading cause of hospital-acquired AKI. It has been reported that oxidative stress associated with superoxide production and related pathways is involved in the pathogenesis of CIN [99], which is modulated by the level of sirtuin expression. We previously demonstrated that SIRT1 activation by resveratrol treatment attenuated CIN via modulation of oxidative stress and apoptosis through activation of PGC-1α/FoxO1 signaling [100]. Another study demonstrated that SIRT3 deficiency aggravated CIN [101], whereas activation of the SIRT3-Nrf2 pathway alleviated CIN [102]. Further investigation into the exact roles of sirtuins in various settings of AKI is warranted.

### 3.2. Sirtuins in the Aging Kidney

Aging is a multifactorial process characterized by progressive decline in physiological function. The kidney is a typical target organ of age-associated tissue damage, and the increased incidence of chronic kidney disease (CKD) in elderly is an emerging health problem worldwide [103]. Various sirtuins have been demonstrated to mitigate kidney aging. SIRT1 expression was found to be reduced in aging kidneys, and this change was associated with changes in the expression of other target molecules such as PGC-1α/estrogen-related receptor-1α (ERR-1α), PPARα, Klotho, and HIF-1α [104,105,106]. Recently, podocyte-specific reduction of SIRT1 was found to accelerate kidney injury in aging mice [107]. Therefore, SIRT1 is believed as a potential target for treatment of kidney aging.

Caloric restriction has been shown to extend lifespan and may affect numerous cellular aspects of kidney aging. Kume et al. demonstrated that long-term caloric restriction from one to two years in mice promoted SIRT1 expression in aging kidneys, which resulted in attenuation of hypoxia-induced kidney injury via SIRT1-mediated deacetylation of FoxO3a and activation of autophagy [12]. Even short-term activation of SIRT1 through caloric restriction promoted autophagy and reduced mitochondrial oxidative damage in 25-month-old rats [108]. SIRT1 and its target proteins may play an important role in renoprotection of aging kidneys, which is accomplished through stimulation of autophagy. Pharmacologically induced SIRT1 activation significantly reduced tubulointerstitial fibrosis and improved renal function through enhancement of Nrf2/HO-1 signaling and AMPK/PGC-1α signaling [109].

Along with other sirtuins, SIRT3 has been reported to act as an essential regulator of cell senescence. SIRT3 is associated with renin–angiotensin–aldosterone system (RAAS) activation, which is known to play a role in kidney aging. In kidneys of aged mice, angiotensin II (Ang II) type 1 receptor (AT_1_R) deletion upregulated nicotinamide phosphoribosyltransferase (Nampt) and SIRT3 and resulted in markedly prolonged lifespan. Ang II treatment downregulated SIRT3 expression in tubular epithelial cells, and this effect was inhibited by AT_1_ antagonist administration. These findings suggest a biochemical link between Ang II and SIRT3 through AT_1_R in aging kidneys [110]. However, a recent study reported conflicting results: this study found that expression of SIRT1 and Nampt expression, but not SIRT3, was significantly reduced in the kidneys of aged mice with AT_1_R-associated protein (ATRAP) deletion [111]. Although the role of SIRT3 in kidney aging via the Ang II-AT_1_R signaling pathway remains unclear, SIRT3 deficiency is known to cause severe renal fibrosis in aging kidneys associated with increased transforming growth factor-β1 (TGF-β1) expression and hyperacetylation of glycogen synthase kinase-3β (GSK-3β), resulting in phosphorylation of Smad3, c-Jun, and β-catenin [112]. SIRT6 activation due to caloric restriction also attenuated age-associated kidney injury through inhibition of the proinflammatory NF-κB signaling pathway [113]. Together, these findings indicate that sirtuins play a role in attenuating tissue injury in aging kidneys, likely via attenuation of oxidative stress and inflammation, and it supports that the investigation of sirtuins should be done as therapeutic targets for kidney aging.

### 3.3. Sirtuins in Diabetic Kidney Disease

Caloric restriction not only slows aging and increases lifespan, but also increases insulin sensitivity [114,115]. In a clinical study, fasting glucose levels and insulin resistance improved after a 12-week intensive weight reduction program based on caloric restriction among obese individuals with advanced diabetic nephropathy (DN), and also led to improvement in kidney function [116]. Dietary restriction in diabetic rat models increased SIRT1 expression in the kidneys and improved renal function including albuminuria, creatinine clearance, and renal histology [117,118]. Thus, caloric restriction activates sirtuins that may be beneficial in preventing the progression of DN.

Several studies have suggested that SIRT1 decreases mitochondrial oxidative stress and apoptosis through modulation of p53 [119], the AMPK/PGC-1α pathway [120,121,122], the Nrf2 pathway [123], and the FoxO family [124], thereby providing protection against DN. SIRT1 also restrained renal inflammation and fibrosis under hyperglycemic conditions through HIF-1α signaling in mesangial cells [125]. Podocyte-specific SIRT1 deletion in diabetic mice led to proteinuria and podocyte injury, and these changes were associated with renal inflammation due to hyperacetylation of STAT3/NF-κB [126]. In addition, SIRT1 modulated angiogenesis through downregulation of vascular endothelial growth factor (VEGF) and Flk-1 (VEGFR-2) expression in high glucose (HG)-treated podocytes and endothelial cells, but these effects were attenuated by the genetic elimination of SIRT1 [127].

SIRT1 may participate in the crosstalk between podocytes and tubular cells in DN. Podocyte-specific SIRT1 deletion caused severe mesangial expansion and podocyte loss [126], while SIRT1 overexpression in podocytes attenuated renal damage in diabetic mice [128]. SIRT1 deletion in proximal tubules also increased albuminuria, which upregulated the tight junction protein claudin-1, in streptozotocin (STZ)-induced diabetic mice [129]. Exposure of podocytes to medium obtained from proximal tubular cells cultured with HG downregulated SIRT1 and upregulated claudin-1 expression. These effects were abolished in podocytes exposed to medium from proximal tubular cells overexpressing SIRT1, even under HG conditions [129]. Based on these findings, Hasegawa et al. proposed a functional relationship between proximal tubules and podocytes, referred to as ‘proximal tubule–podocyte communication’ [129].

Recent research has demonstrated a role for SIRT1 in proximal tubule–podocyte communication in association with sodium–glucose cotransporter 2 (SGLT2). In diabetic kidneys, high glucose levels around the proximal tubules may trigger glucose transporter 2 (GLUT2)-mediated intracellular glucose uptake via SGLT2 upregulation, causing a concomitant decrease in SIRT1. An SGLT2 inhibitor recovered SIRT1 expression in diabetic mice and HG-treated proximal tubular cells [130], as well as AMPK phosphorylation [131]. Interplay between AMPK/SIRT1 signaling and sodium transport mechanisms in the kidney may partially explain the role of the SGLT2 inhibitor in ameliorating the development of DN [132].

The associations of sirtuins other than SIRT1 with DN have also been explored. SIRT3 overexpression suppressed HG-induced apoptosis by reducing ROS accumulation through modulation of Akt/FoxO signaling in proximal tubular cells [133]. On the other hand, SIRT3 suppression was associated with activation of TGF-β/Smad3 signaling and increased HIF-1α accumulation, which subsequently caused abnormal glycolysis and kidney fibrosis in diabetic mice and proximal tubular cells [134]. SIRT4 overexpression led to downregulated expression of apoptosis-related proteins such as NADPH oxidase 1 (NOX1), Bcl-2-associated X protein (Bax), and phosphorylated p38, along with upregulated expression of Bcl-2, which was associated with attenuation of the inflammatory response in HG-simulated podocytes [135].

SIRT6 deletion exacerbated podocyte injury in diabetic mice, and SIRT6 overexpression with HG treatment protected against podocyte injury through epigenetic regulation of Notch1 and Notch4 transcription due to deacetylation of H3K9 [73]. SIRT6 was also found to regulate the immune response by activating M2 macrophages, which are protective against podocyte injury, in STZ-induced diabetic mice [136]. In a recent study, selective deletion of Nampt in proximal tubule cells of STZ-induced diabetic mice led to downregulation of SIRT6, which was accompanied by thickening of the tubular basement membrane, type IV collagen deposition, enhanced renal fibrosis, and albuminuria. Selective deletion of SIRT6 in the proximal tubules of diabetic mice caused a phenotype similar to that of Nampt knockout mice. Therefore, the Nampt–Sirt6 axis in proximal tubules was suggested to be a key player in the fibrogenic extracellular matrix remodeling associated with DN [137].

### 3.4. Sirtuins in Chronic Kidney Disease

Renal tubular fibrosis is a major pathognomonic phenomenon in CKD [138], and sirtuins have been demonstrated to play an important role in tubular fibrosis. SIRT1 knockout mice were found to have prominent tubular fibrosis in a model of unilateral ureter obstruction (UUO) [139]. Suppression of SIRT1 expression in mouse renal medullary interstitial cells resulted in substantial reduction of cellular resistance to oxidative stress [139]. In addition, SIRT1 activation inhibited tubular fibrosis in a 5/6 nephrectomy model and a UUO model [140,141]. SIRT1 expression was also found to be involved in the pathogenesis of chronic renal allograft dysfunction and chronic cyclosporine A (CsA) nephropathy. In rat kidneys with chronic allograft dysfunction, decreased SIRT1 was associated with mononuclear cell infiltration and interstitial fibrosis due to upregulation of inflammatory cytokines [142]. In a mouse model of chronic CsA nephropathy, SIRT1 expression was reduced according to the degree of tubulointerstitial fibrosis through the Nrf2 and PI3K/Akt/FoxO1 pathways [143].

Various mechanisms have been suggested to underlie the pathogenetic link between SIRT1 and the development of renal fibrosis. Endothelial SIRT1 expression may play an important role, as SIRT1 deletion in the endothelium of mice caused spontaneous interstitial fibrosis without glomerular involvement, even at a young age. Moreover, tubulointerstitial fibrosis after long-term folic acid treatment was aggravated in mice with endothelium-specific SIRT1 deletion [144]. Endothelial SIRT1 depletion also enhanced the senescence of pericapillary tubular endothelial cells, which manifested as impaired endothelial proliferation and increased expression of molecules in the Notch1 signaling pathway [145]. A role for sirtuins in the epithelial-to-mesenchymal transition (EMT) during the development of renal fibrosis has also been suggested [146]. SIRT1 up-regulation by resveratrol treatment ameliorated renal fibrosis in proximal tubular cells treated with TGF-β in addition to a mouse model of UUO; this was found to be due to inhibition of the EMT through deacetylation of Smad4 and inhibition of matrix metalloproteinase-7 (MMP-7) [147].

Consistent with observations in AKI, pharmacological inhibition of SIRT2 resulted in reduction of renal interstitial fibrosis in UUO models [148,149], which was accompanied by decreases in expression of epidermal growth factor receptor (EGFR), platelet-derived growth factor receptor-β (PDGFR-β), STAT3 [148], and E3-ubiquitin ligase murine double-minute 2 (MDM2) [149]. In contrast, SIRT3 plays an important role in the endothelial-to-mesenchymal transition (EndoMT) associated with the vascular pathology of renal fibrosis [150]. EndoMT is a novel mechanism of renal fibrosis and is characterized by a phenotypic transition from vascular endothelial cells to myofibroblasts [151]. Honokiol, a pharmaceutical SIRT3 activator, decreased renal inflammation and fibrosis through regulation of mitochondrial dynamics via the NF-κB/TGF-β1/Smad signaling pathway [152]. SIRT6 knockout aggravated TGF-β-induced fibrosis in mouse tubular epithelial cells, while pharmacological inhibition of SIRT6 deacetylase activity by OSS_128167 induced kidney fibrosis in a mouse model of UUO through modulation of the Wnt/β-catenin signaling pathway [74]. Therefore, various sirtuins appear to be involved in kidney fibrosis and related processes.

## 4. Summary and Future Perspectives

**Figure 1:**
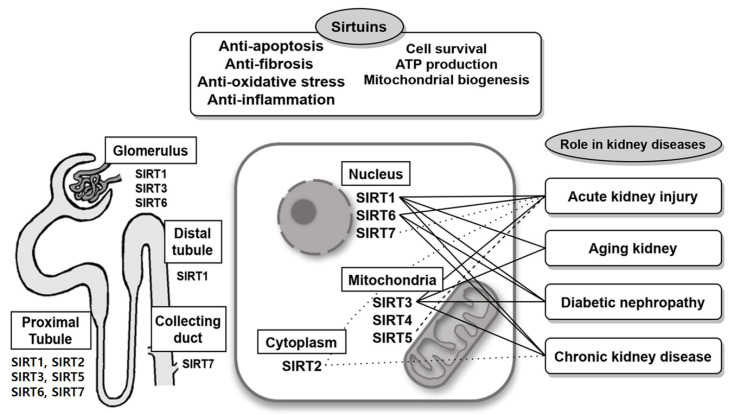
Implication of sirtuins in various kidney diseases associated with variable cellular location of sirtuins and associated pathways. The types of lines (solid line: attenuation of disease, dotted line: aggravation of disease, dash line: controversial) between each sirtuin with kidney disease were based on previous studies.

Sirtuins play critical roles in cellular homeostasis, and numerous published studies have revealed that sirtuins participate in various acute and chronic kidney diseases through the regulation of oxidative stress, apoptosis, inflammation, fibrosis, cell survival, ATP production and mitochondrial biogenesis (Figure 1, Table 1). Considering the fact that the functions of sirtuins have been well characterized in animal models, more research into the role of these proteins in human kidney diseases is warranted. Development of kidney-specific sirtuin activators will facilitate further investigation of sirtuins as novel therapeutic targets.

## Figures and Tables

**Table 1:** Pathophysiologic role of sirtuins in kidney diseases (↓: decreases, ↑: increases).

Experimental Models		Interventions	Renal Outcome/Phenotype	Mechanism	Ref
**SIRT1**					
**AKI**	I/R injury	SRT-1720	Apoptosis ↓	SIRT1 ↑ → acetylated p53 ↓	[81]
		SRT-1720	Mitochondrial biogenesis ↑	SIRT1 ↑ → PPARγ, ATP levels ↑ SIRT1 ↑ → deacetylated PGC-1α ↑	[11] [83]
	Cisplatin	Tubule-specific SIRT1 Tg	Apoptosis and ROS production ↓ Function and number of mitochondria and peroxisome ↑	SIRT1 ↑ → catalase, PGC-1α ↑	[87]
		SRT-1720	Apoptosis and inflammation ↓	SIRT1 ↑ → acetylated p53, NF-κB, TNF-α ↓	[88]
	Sepsis	Resveratrol	Oxidative stress ↓, mitochondrial function ↑	SIRT1 ↑ → acetylated SOD2 ↓	[98]
	Contrast	Resveratrol	Oxidative stress ↓	SIRT1 ↑ → PGC-1α ↑, FoxO1 ↑	[100]
**Aging**	Aging mice	Doxycycline-inducible podocyte-specific RNAi model for SIRT1 (Pod-SIRT1 RNAi)	Oxidative stress ↑	SIRT1 ↓ → PGC-1α ↓, PPARγ ↓, acetylated FoxO3a, FoxO4, NF-κB ↑	[107]
	Aging mice	Resveratrol	Inflammation ↓	SIRT1 ↑ → Nrf2/HO-1 ↑, AMPK/PGC-1α ↑	[109]
**DKD**	STZ-diabetic rats	Resveratrol	Apoptosis and oxidative stress ↓ Angiogenesis ↓	SIRT1 ↑ → acetylated p53 ↓ SIRT1 ↑ → Nrf2 ↑ SIRT1 ↑ → phosphorylated FoxO1 ↓ SIRT1 ↑ → VEGF and Flk-1 ↓	[119] [123] [124] [127]
	*db/db* mice	Resveratrol	Mitochondrial biogenesis ↑ Oxidative stress and apoptosis ↓	SIRT1 ↑ → Mn-SOD↑, AMPK ↑ SIRT1 ↑ → AMPK/PGC-1α ↑, PPARα ↑	[120] [121] [122]
	*db/db* mice	Podocyte-specific SIRT1 KO (Podocin-Cre; SIRT1^fl/fl^) Proximal tubule-specific SIRT1 KO	Inflammation and apoptosis ↑ Albuminuria and podocyte injury ↑	SIRT1 ↓ → acetylated p65, STAT3, FoxO4 ↑ SIRT1 ↓ → claudin-1 ↑	[126] [129]
	OVE26 mice	Podocyte-specific SIRT1 overexpression (Podocin-SIRT1^OV^)	Podocyte injury ↓	SIRT1 ↑ → PGC-1α ↑	[128]
**CKD**	UUO	SIRT1 KO in endothelium (Tie2-Cre with SIRT1^F/F^)	Apoptosis and fibrosis ↑ Senescence of peritubular capillary ECs ↑	SIRT1 ↓ → renal COX2 ↓ SIRT1 ↓ → Notch1 ↑	[139] [145]
	UUO	Resveratrol	Interstitial fibrosis ↓, EMT ↓	SIRT1 ↑ → acetylated Smad3 ↓ SIRT1 ↑ → MMP-7 ↓	[140] [147]
	UUO	SIRTinol, EX527	Renal fibrosis ↓	SIRT1, SIRT2 ↓ → EGFR ↓, PDGFR ↓	[148]
	5/6 nephrectomized mice	SIRT1 KO	Interstitial fibrosis ↑	SIRT1 ↓ → acetylated Smad3 ↑	[141]
	Folic acid nephropathy	Endothelial-deleted SIRT1^endo-/-^	Fibrotic response, angiogenesis ↑	SIRT1 ↓ → MMP-14 ↓	[144]
**SIRT2**					
**AKI**	Cisplatin	SIRT2 KO	Apoptosis, necroptosis, inflammation ↓	SIRT2 ↓ → MKP-1 ↑ → p38, JNK ↓	[90]
	Sepsis	Genetic deletion of SIRT2^-/-^	Inflammation ↓	SIRT2 ↓ → CXCL2 ↓, CCL2 ↓, MKP-1 ↑ → p38, JNK ↓	[34]
**CKD**	UUO	AGK2	Tubulointerstitial fibrosis↓	SIRT2 ↓ → MDM2-p53 ↓	[149]
	UUO	AGK2	Renal fibrosis ↓	SIRT1, SIRT2 ↓ → EGFR ↓, PDGFR ↓	[148]
**SIRT3**					
**AKI**	Cisplatin	Genetic deletion of SIRT3^-/-^	Oxidative stress ↑, mitochondrial function ↓	SIRT3 ↓ → DRP1 ↑	[91]
	Contrast	Genetic deletion of SIRT3^-/-^	Oxidative stress and apoptosis ↑	SIRT3 ↓ → MnSOD, catalase ↓	[101]
**Aging**	Aging mice	SIRT3 KO	Renal fibrosis ↑	SIRT3 ↓ → acetylated GSK-3β ↑ → Smad3, c-Jun, and β-catenin ↑	[112]
**DKD**	STZ-diabetic mice	SIRT3 siRNA	Fibrosis and aberrant glycolysis ↑	SIRT3 ↓ → TGF-β/smad3 ↑, HIF-1α ↑	[134]
**CKD**	UUO	SIRT3 endothelial cell-specific Tg (SIRT3-Tg^EC^)	Renal fibrosis, EndoMT ↓, Oxidative stress ↓	SIRT3 ↑ → FoxO3a nuclear translocation ↑	[150]
	UUO	Honokiol	Mitochondrial fusion ↑, Inflammation↓	SIRT3 ↑ → NF-κB/TGF-β1/Smad ↓	[152]
**SIRT5**					
**AKI**	I/R injury, Cisplatin	Genetic deletion of SIRT5^-/-^	Peroxisomal fatty acid oxidation ↑	SIRT5 ↓ → mtDNA ↑	[65]
**SIRT6**					
**AKI**	Cisplatin	SIRT6 Tg	Apoptosis ↓	SIRT6 ↑ → ERK1/2 ↓	[94]
**DKD**	STZ-treated uninephrectomized mice	Podocyte-specific SIRT6 KO (Podocin-Cre^+^/SIRT6^fl/fl^)	Podocyte injury ↑, Autophagy ↓	SIRT6 ↓ → acetylated H3K9 ↑, Notch1/4 ↑	[73]
	STZ-diabetic rats	SIRT6 overexpression	Podocyte injury ↓	SIRT6 ↑ → M2 macrophage ↑	[136]
**CKD**	UUO	OSS_128167	Renal fibrosis ↑	SIRT6 ↓ → β-catenin ↑, acetylated H3K56 ↑	[74]
**SIRT7**					
**AKI**	Cisplatin	Genetic deletion of SIRT7^-/-^	Apoptosis, oxidative stress, inflammation ↓	SIRT7 ↓ → TNF-α ↓, nuclear NF-κB ↓	[79]

Abbreviations: AKI, acute kidney injury; AMPK, adenosine monophosphate-activated protein kinase; ATP, adenosine triphosphate; CCL2, C-C motif chemokine ligand 2; CKD, chronic kidney disease; COX2, cyclooxygenase-2; CXCL2, C-X-C motif chemokine ligand 2; DKD, diabetic kidney disease; DRP1, dynamin related protein; Endo or EC, endothelial cell; EGFR, epidermal growth factor receptor; EMT, epithelial-to-mesenchymal transition; EndoMT, endothelial-to-mesenchymal transition; ERK, extracellular-signal-regulated kinase; FoxO, forkhead box O; GSK-3β, glycogen synthase kinase-3β; HIF-1α, hypoxia-inducible factor-1α; HO-1, heme oxygenase-1; I/R, ischemia/reperfusion; JNK, c-Jun N-terminal kinase; KO, knockout; MDM2, E3-ubiquitin ligase murine double-minute 2; MKP-1, Mitogen-activated protein kinase-1; MMP, Matrix metalloproteinase; NF-κB, Nuclear factor-kappa B; NOX, NADPH oxidase; Nrf2, nuclear factor erythroid 2–related factor 2; mtDNA, mitochondrial DNA; NF-κB, nuclear factor-kappa B; OV, overexpression; PDGFR, Platelet-derived growth factor receptor; PGC-1α, peroxisome proliferator–activated receptor-γ coactivator 1α; PPAR, peroxisome proliferator-activated receptor; Pod, podocyte; siRNA, small interfering RNA; SIRT, sirtuin; SOD, superoxide dismutase; STAT3, Signal transducer and activator of transcription 3; STZ, streptozotocin; Tg, transgenic; TGF-β1, transforming growth factor-β1; TNF-α, tumor necrosis factor-α; UUO, unilateral ureteral obstruction; VEGF, Vascular endothelial growth factor.

## Abbreviations

ACSS2	Acetyl-coenzyme A synthetase 2
ADP	Adenosine diphosphate
AKI	Acute kidney injury
AMPK	Adenosine monophosphate-activated protein kinase
Ang II	Angiotensin II
AT_1_R	Angiotensin II type 1 receptor
ATP	Adenosine triphosphate
ATRAP	AT_1_R-associated protein
Bax	Bcl-2-associated X protein
Bcl-2	B-cell lymphoma 2
CCL2	C-C motif chemokine ligand 2
CIN	Contrast-induced nephropathy
CKD	Chronic kidney disease
COX2	Cyclooxygenase-2
CsA	Cyclosporine A
CXCL2	C-X-C motif chemokine ligand 2
DKD	Diabetic kidney disease
DN	Diabetic nephropathy
DRP1	Dynamin related protein 1
EGFR	Epidermal growth factor receptor
EMT	Epithelial-to-mesenchymal transition
EndoMT	Endothelial-to-mesenchymal transition
ERK	Extracellular-signal-regulated kinase
ERR-1α	Estrogen related receptor-1α
FoxO	Forkhead box O
GABP-β1	GA-binding protein-β-1
GLUT2	Glucose transporter 2
GSK-3β	Glycogen synthase kinase-3β
HDACs	Histone deacetylases
HG	High glucose
HIF-1α	Hypoxia-inducible factor-1α
HO-1	Heme oxygenase-1
I/R	Ischemia/reperfusion
JNK	c-Jun N-terminal kinase
LKB1	Liver kinase B1
MCD	Malonyl CoA decarboxylase
MDM2	E3-ubiquitin ligase murine double-minute 2
MKP-1	Mitogen-activated protein kinase-1
MMP	Matrix metalloproteinase
mtDNA	Mitochondrial DNA
mTOR	Mammalian target of rapamycin
NAD	Nicotinamide adenine dinucleotide
Nampt	Nicotinamide phosphoribosyltransferase
NF-κB	Nuclear factor-kappa B
NOX	NADPH oxidase
NPM1	Nucleolar protein nucleophosmin
Nrf2	Nuclear factor erythroid 2–related factor 2
OXPHOS	Oxidative phosphorylation
PAF53	Polymerase-associated factor 53
PDGFR-β	Platelet-derived growth factor receptor-β
PEPCK1	Phosphoenolpyruvate carboxykinase 1
PGC-1α	Peroxisome proliferator–activated receptor-γ coactivator 1α
PI3K	Phosphatidylinositol-3-kinase
PPAR	Peroxisome proliferator-activated receptor
RAAS	Renin–angiotensin–aldosterone system
ROS	Reactive oxygen species
SGLT2	Sodium–glucose cotransporter 2
SIR2	Silent information regulator 2
SIRT	Sirtuin
SOD	Superoxide dismutase
STAT3	Signal transducer and activator of transcription 3
STZ	Streptozotocin
TGF-β1	Transforming growth factor-β1
TNF-α	Tumor necrosis factor-α
UUO	Unilateral ureter obstruction
VEGF	Vascular endothelial growth factor
